# Manual lymphatic drainage adds no further volume reduction to Complete Decongestive Therapy on breast cancer-related lymphoedema: a multicentre, randomised, single-blind trial

**DOI:** 10.1038/s41416-018-0306-4

**Published:** 2018-10-24

**Authors:** Mette Tambour, Marianne Holt, Anette Speyer, Robin Christensen, Bibi Gram

**Affiliations:** 1 Department of Physiotherapy and Occupational Therapy, Hospital of Southwest Jutland, Esbjerg, Denmark; 2 Department of Rehabilitation, Odense University Hospital, Odense, Denmark; 3 Department of Physiotherapy and Occupational Therapy, Lillebaelt Hospital, Vejle, Denmark; 4 Musculoskeletal Statistics Unit, The Parker Institute, Bispebjerg and Frederiksberg Hospital, Copenhagen, Denmark; 5 Department of Rheumatology, Odense University Hospital, Odense, Denmark; 6 Research Unit of Health Science, Hospital of Southwest Jutland, Esbjerg and Department of Regional Health Research, University of Southern Denmark, Odense, Denmark

**Keywords:** Rehabilitation, Breast cancer

## Abstract

**Background:**

We investigated the comparability of Complete Decongestive Therapy (CDT) including manual lymphatic drainage (MLD) vs. without MLD in the management of arm lymphoedema in patients with breast cancer.

**Methods:**

Patients randomised into either treatment including MLD (T+MLD) or treatment without MLD (T−MLD) received treatment 2×weekly for 4 weeks. The primary outcome was the volume reduction (%) of arm lymphoedema at 7-month follow-up. The secondary outcomes were volume reduction after the end of treatment, circumference of the arm, patient experience of heaviness and tension, and health status.

**Results:**

Despite difficulties enrolling the planned number of patients (160), 77 were randomised and 73 (38 in T+MLD, 35 in T−MLD) completed the trial. In both groups, the volume of lymphoedema decreased significantly, with no difference between groups (1.0% [95% CI, −4.3;2.3%]): the precision in the 95% confidence interval indicates that the efficacy was comparable; the mean (SE) changes at month 7 were −6.8%(1.2) and −5.7% (1.2) in the T+MLD and T−MLD, respectively. There were no statistically significant differences with respect to any of the secondary outcomes. The results were robust and the conclusion was not sensitive even to various alternative assumptions or analytic approaches to data analysis.

**Conclusion:**

Manual lymphatic drainage adds no further volume reduction in breast cancer patients.

## Introduction

A well-known complication of breast cancer treatment is secondary lymphoedema. According to ‘Breast Cancer Statistics’, nearly 1.7 million new cases of breast cancer were diagnosed worldwide in 2012.^1^ In Denmark, 4810 women were diagnosed with breast cancer in 2016.^2^ The incidence of lymphoedema among breast cancer survivors is ambiguous and depends on the type of surgery received.^3–5^ The women diagnosed with lymphoedema at the hospital are referred to physiotherapy. If the circumferential measurement of lymphoedema in the affected upper extremity is 2 cm above the unaffected upper extremity in at least one measurement and/or by another reason assessed by the physiotherapist, the patients are generally offered ‘Complete Decongestive Therapy’ (CDT). CDT is a concept of treatment consisting of skin care, manual lymphatic drainage (MLD), bandaging, and exercise. The most commonly used bandages in CDT are so-called short-stretch bandages. These must be changed several times a week; however, over the years new bandages have been developed that may lead to other treatment strategies. By using, for example Coban^TM^ Lite bandages, it is no longer necessary to change the bandages more than twice a week.^6^ Traditionally, patients with lymphoedema are offered all four components of CDT, and by using short-stretch bandages, the treatment often requires logistics management and detailed implementation, which can be very time-consuming.^7–9^ Meanwhile, the development of new improved bandages in combination with a lack of scientific evidence of MLD challenge the existing structure of CDT regarding the use of the type of bandages, the content of the treatment, and thus the time spent on each treatment. New treatment strategies might need to be considered to optimise the treatment efficiency and one solution might be to change the type of bandages and/or to exclude one of the CDT components.

Andersen et al.^10^ showed no difference between standard therapy and MLD added to standard therapy. Standard therapy consisted of a custom-made sleeve and glove and educational instruction in physical exercises, skin care, and safety precautions. The primary outcome was a reduction in absolute oedema volume calculated from arm circumferences measurement. In a randomised non-inferiority trial, Gradalski et al.^11^ found that MLD might not be necessary in CDT, since it did not reduce the volume of lymphoedema significantly. This is in line with results from a systematic review^12^ and Cochrane review,^13^ which also emphasised that there was no scientific evidence on between-group differences in outcomes such as pain, heaviness, and quality of life. However, both reviews underline that the randomised control trials (RCTs) included in the analyses suffer from clinical and statistical inconsistencies. According to the reviews, the methodological quality of the evidence varied among the trials and the conclusion is that more high-quality studies are warranted.

Therefore, our purpose was to design a methodologically rigorous trial critically assessing the impact of the ongoing practice in the treatment of breast cancer patients with upper extremity lymphoedema. The aim was to investigate the effectiveness of CDT with MLD compared to a treatment with three of CDT components and without MLD, when treating arm lymphoedema in patients with breast cancer. Our hypothesis was that CDT is equally effective whether it includes MLD or not.

## Methods

### Study design and setting

This study was designed as a randomised, single-blind, equivalence trial of two different composite physiotherapy treatments for lymphoedema after breast cancer surgery. Both management strategies were based on the principles of CDT. Patients with lymphoedema were recruited from three hospitals in the Region of Southern Denmark: Lillebaelt Hospital/Vejle, Odense University Hospital, and Hospital of Southwest Jutland, Esbjerg.

All the patients received oral and written information about the study and written informed consent forms were obtained prior enrolment in the study. Patients who did not wish to participate in the study received standard treatment for their lymphoedema. The study was registered with The Danish Data Protection Agency, in ClinicalTrials.gov (NCT02015897) http://www.clinicaltrials.gov/, conducted according to the Declaration of Helsinki, and approved by The Regional Committees on Health Research Ethics for Southern Denmark (S-20130091).

### Patients

Patients eligible for this study were included from January 2014 to April 2017. Patients were referred from oncologists or breast surgeons and were assessed for eligibility by a physiotherapist specialised in the field. At each centre, the included patients were randomised (1:1) using random block sizes of 2, 4, and 6 into group who received all four components of CDT: treatment included MLD (T+MLD) or into group who received treatment with three of CDT components and without MLD (T−MLD).

This study's inclusion criteria were: unilateral breast cancer diagnosis regardless of the date of operation and identified lymphoedema, lymphoedema >2 cm and in stage ll–lll,^14^ ultrasound scanning of the axilla in order to exclude local relapse, completed radiotherapy, and/or chemotherapy at least 6 weeks prior inclusion. The latter criterion was initially meant to be 2 months prior inclusion^15^; however, this was changed 6 weeks after the start of the project. The exclusion criteria were: relapse of breast cancer, untreated infection, untreated heart failure, untreated renal failure, untreated deep venous thrombosis in the arm, inability to participate in physiotherapy treatments, and/or inability to understand the instructions. More details are available in the published protocol.^15^

### Interventions

Details on the intervention have been reported elsewhere.^15^ Briefly, the T+MLD group received treatment including the four components of CDT: (1) skin care, (2) MLD according to Földi's technique,^7–9^ (3) bandaging using Coban^TM^ 2 Lite, and (4) guidance on physical activity. Coban^TM^ 2 Lite is a two-component compression system with a comfort layer close to the skin and a self-adherent external compression layer. The bandage that is designed to give pressure at 20–30 mm Hg was retained until the next treatment. This treatment is in accordance with what is offered at the hospitals to similar patients as those meeting this study’s inclusion criteria. The T−MLD group received treatment consisting of the same components except from MLD. The intervention was given twice a week for 30 or 60 min, depending on which group the patients were randomised to. Omission of MLD was expected to reduce the treatment time.

The intervention period for both groups was approximately 3 weeks. At the time, two consecutive measurements were stationary (<0.5 cm difference in 3 out of the 5 measurements), a permanent and individually tailored compression sleeve was ordered. All patients received treatment while waiting for the compression sleeve (about one week). After around 4 weeks, the sleeve was handed to the patients and they were instructed in how to apply it and were recommended to use the sleeve every day during daytime and to remove it only at bedtime. All in all, the treatment period lasted for roughly 4 weeks followed by a 6-month follow-up period.^15^

### Tests and outcome measures

All measurements were standardised and performed 3 times: before randomisation (baseline), after ended treatment (1 month), and at follow-up (7 months).^15^ The test personnel were blinded to the patients’ group allocation.

The primary outcome was the percentage total volume reduction of lymphoedema from baseline to 7 months. The lymphoedema volume (ml) was measured by Inverse Water Displacement Volumetry method^16^ using Bravometer (Novuqare BV, PJ Horst, NL). Bravometer is an apparatus developed to measure the shortness of water after the patient's arm has been moved from the Bravometer. The method was validated by Damstra in 2006.^17^

*Total volume* was defined as the lymphoedema volume in the affected arm only. *Excess volume* was defined as the lymphoedema volume where the affected limb was compared to the unaffected limb by subtracting the unaffected arm from the affected arm.^13^ The relative reduction (%) of excess volume was calculated as:

$${\displaystyle{{\textstyle ({\mathrm{post - volume}}\,{\mathrm{in}}\,{\mathrm{affected}}\,{\mathrm{arm}}-{\mathrm{post - volume}}\,{\mathrm{in}}\,{\mathrm{unaffected}}\,{\mathrm{arm}})\hfill \atop\\ \textstyle -({\mathrm{pre - volume}}\,{\mathrm{in}}\,{\mathrm{affected}}\,{\mathrm{arm}}-{\mathrm{pre - volume}}\,{\mathrm{in}}\,{\mathrm{unaffected}}\,{\mathrm{arm}})\hfill } \over {({\mathrm{pre - volume}}\,{\mathrm{in}}\,{\mathrm{affected}}\,{\mathrm{arm}}-{\mathrm{pre - volume}}\,{\mathrm{in}}\,{\mathrm{unaffected}}\,{\mathrm{arm}})}}} \ast 100$$

The secondary outcome measurement included percentage of volume reduction of lymphoedema from baseline to after treatment (short-term effect) and a reduction in the measured circumference of the affected arm (cm) based on five predetermined points on the arm and two points on the hand. The sum of the seven measurements was calculated, and the short-term difference and the difference from baseline to follow-up were evaluated.

Patient sensation of heaviness and tensions in shoulder, arm, and chest wall was registered on a numeric rating scale from 0 to 10 (0 = no heaviness, 10 = worst imaginable heaviness). Furthermore, self-reported levels of mobility, pain, anxiety, and quality of life were tested using the questionnaire EQ-5D-5L.^18,19^ Additionally, the patients were asked at follow-up how frequently (on average) they used the sleeve (daily, >3 times per week, ≤1 time per week, or never).

### Statistical methods

The predefined margin of equivalence was ±12% for percentage of the volume level in relation to between-group comparison. This limit was defined as an acceptable range of imprecision.^10,20^ With 95% confidence interval (CI) bounds of −12 and +12% for the mean difference and a significance level of 0.05, assuming a null difference and a common standard deviation of 25%,^20,21^ a two-sided test analysis for additive equivalence of two-sample normal means showed a required a total sample size of 152 patients. It was decided to enrol 160 participants in total.

Baseline demographic and clinical characteristics were reported descriptively for all patients in the full analysis set. Normally distributed characteristics were presented as means ± SD or in medians with interquartile range (IQR). The analysis of the primary outcome was performed according to the intention-to-treat principle. To evaluate the clinical effectiveness, in the form of longitudinal response to the interventions, all outcome variables were analysed using a multilevel repeated-measure mixed effects model with participant ID as the random effect factor (based on a restricted maximum likelihood estimate). The outcome variable was used as the dependent variable, adjusting for the level at baseline (as a covariate) and categorical index variables time and group were handled as class variables. An interaction term of group×time was included. Data from both assessed time points (after baseline) were included. This was modelled using PROC MIXED in the SAS System: this involves modelling variation between participants and covariation between measures at two different times on the same patients.

## Results

**Fig. 1:**
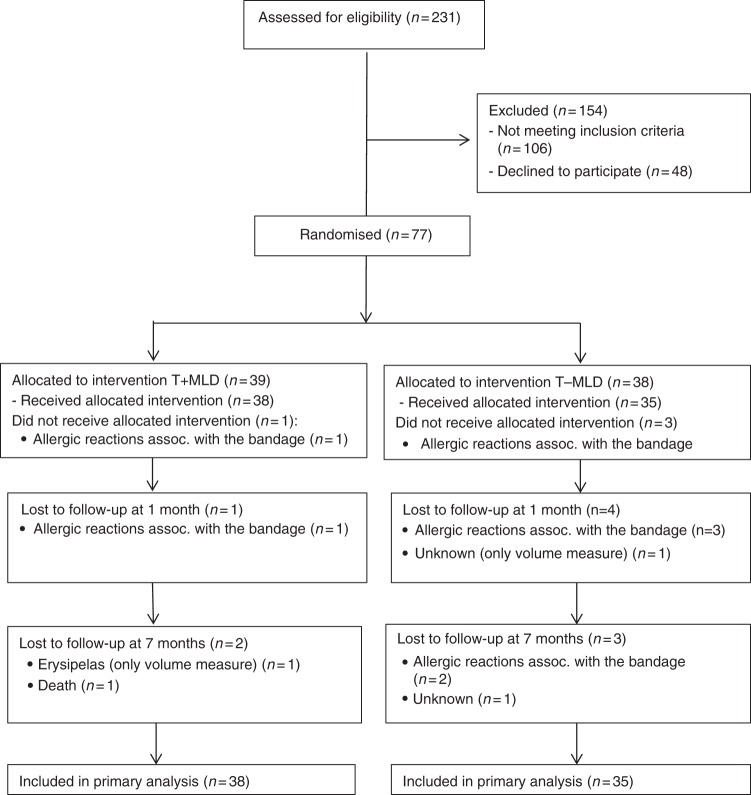
Flow chart of the study ‘Manual lymphatic drainage adds no further volume reduction to Complete Decongestive Therapy on breast cancer-related lymphoedema: a multicentre, randomised, single-blind trial

Despite our prospective sample size estimation, we had difficulties enrolling the 160 patients. We were only able to enrol 77 participants—breast cancer patients with lymphoedema—into the study (which would in a prospective equivalence trial setting correspond to around 35% power). During the study, we expanded the recruitment period two times; however, when we realised that all centre had slow recruitment we decided for ethical reasons to close the recruitment in April 2017. Out of the 77 included patients, 5 patients did not complete test two and 2 of these did not complete test three either. A total of four patients were lost at follow-up (Fig. 1). These four were not significantly different from those who completed the study in any variables (data not shown). The inclusion criterion regarding the timeframe of completed radiotherapy was changed during the project from 2 months to 6 weeks, since no clinically significant arguments were against shortening the timeframe. The modification was done only 6 months after the start of the trial and at that time only 10 patients were enrolled into the study.

### Clinical characteristics

Clinical characteristics for the study sample are presented in Table 1. As expected from the randomisation, the groups appeared comparable for all major baseline characteristics (Table 1). The timeframe from surgery to onset of lymphoedema was median (IQR) 12 (5;33) months in T+MLD and 11 (4;24) months in T−MLD. Between onset of lymphoedema and starting the treatment, the time span was median (IQR) 6 (3;15) months and 6 (4;16) months in T+MLD and T−MLD, respectively.

**Table 1:** Baseline demographic and clinical characteristics

Characteristics	Treatment Incl. MLD		Treatment+no MLD			Total (all)		
	*N*	Mean (SD)	*N*	Mean (SD)	*N*	Mean	Min.	Max.
Age (years)	39	62.0 (11.5)	38	60.9 (10.8)	77	61.5 (11.1)	32	83
Height (m)	39	1.7 (0.1)	38	1.6 (0.1)	77	1.6 (0.1)	1.45	1.8
Body weight (kg)	39	80.4 (14.7)	38	79.7 (14.5)	77	80.0 (14.5)	57.3	120.5
BMI (kg/m^2^)	39	29.6 (5.5)	38	29.4 (5.3)	77	29.5 (5.3)	21.6	41.7
Dominant hand, right/left (*n*)	39	35/4	38	38/0	77	73/4		
Affected arm, right/left (*n*)	39	17/22	38	19/19	77	36/41		
Primary outcome								
Total volume in the affected arm (ml)	39	2942.9 (593.9)	38	2889.7 (491.3)	77	2916.6 (542.8)	2047	4690
Secondary outcomes								
Excess volume (ml)	39	489.4 (298.2)	38	485.3 (305.9)	77	487.4 (300.1)	−138	1301
Total volume in the unaffected arm (cm)	39	2453.5 (441.4)	38	2404.4 (460.1)	77	2429.3 (448.4)	1610	3725
Circumference in the affected arm (cm)	39	184.3 (17.2)	38	182.1 (14.1)	77	183.2 (15.6)	152.8	226
Circumference in the unaffected arm (cm)	39	154.9 (26.8)	38	153.5 (23.2)	77	154.2 (24.9)	113.7	213
Health today (VAS: 0–100)	39	75.8 (16.5)	37	69.8 (17.8)	76	72.9 (17.3)	30	100

	*N*	Median (IQR)	*N*	Median (IQR)	*N*	Median (IQR)	Min.	Max.
Time from surgery to oedema (months)	39	12 (5; 33)	38	11 (4; 24)	77	12 (5; 24)	0	156
Time from oedema to treatment (months)	39	6 (3; 15)	38	6 (4; 16)	77	6 (4; 15)	0	158
Patients' experience of								
Heaviness in the arm (scale, 0–10)	37	4 (2; 7)	37	5 (3; 6)	74	5 (3; 7)	0	9
Tension in the arm (scale, 0–10)	37	3 (2; 6)	37	5 (3; 7)	74	4 (2; 6)	0	9
Tension in the shoulder (scale, 0–10)	37	3 (0; 6)	37	3 (0; 5)	74	3 (0; 6)	0	10
Tension in the chest (scale 0–10)	37	4 (2; 7)	37	4 (3; 8)	74	4 (3; 7)	0	10
Mobility (score 1–5)	39	1 (1; 2)	37	1 (1; 1)	76	1 (1; 1)	1	3
Self-care (score 1–5)	39	1 (1; 1)	37	1 (1; 1)	76	1 (1; 1)	1	3
Usual activities (score 1–5)	39	2 (1; 3)	37	2 (2; 3)	76	2 (1; 3)	1	5
Pain (score 1–5)	39	2 (1; 3)	37	2 (2; 3)	76	2 (2; 3)	1	4
Anxiety (score 1–5)	39	1 (1; 2)	37	1 (1; 2)	76	1 (1; 2)	1	4

*SD* standard deviation, *IQR* interquartile range

Fifty percent of the patients underwent mastectomies (*n* = 38) and 50% had a lumpectomy. One patient (*n* = 1) was operated in the axilla only since no primary tumour was found. Only one of the women included had ‘sentinel node removal’, whereas 75 patients had ‘total lymph node removal’ and there was no information on the type of surgery for 1 patient (*n* = 1). Ninety-seven percent of the women (*n* = 75) received radiation therapy post-surgery and 66% had chemotherapy (T+MLD: *n* = 26, T−MLD: *n* = 25). There was no difference with respect to which arm the lymphoedema occurred: in T+MLD, the distribution was (right/left): 22/17 and in T−MLD, 19/19. In both groups, there were 19 patients having lymphoedema in their dominant arm. No difference was observed at baseline between the dominant left and right arm affected by lymphoedema. Regarding the severity of the excess volume, categories have been defined as mild:<20% excess limb volume, moderate: 20–40% excess limb volume, and severe:>40% excess limb volume.^14^ Based on these classifications, 57% (*n* = 44) of the patients in the present study were classified with mild lymphoedema, 43 of the patients (*n* = 32) had moderate lymphoedema, and no patients had severe lymphoedema.

### Primary outcome

**Fig. 2:**
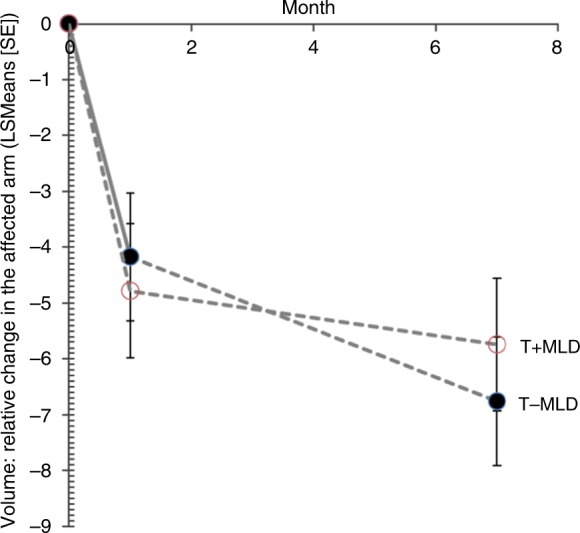
The primary outcome, lymphoedema volume, after 1 month and after 7 months

As reported in Table 2, after the treatment, the volume of lymphoedema decreased significantly in both groups confirming that both treatments were effective, but no statistically significant difference was found between T+MLD and T−MLD (mean ± SE): ∆: 25.06 ml (95% CI, −80.15 to 130.27 ml). As illustrated in Fig. 2, at follow-up, the relative volume reduction at follow-up was (mean ± SE): −1.02 ± 1.66 ml (95% CI, −4.30 to −2.26), corresponding to a statistically insignificant difference between the groups, *p* = 0.540, which confirms that the two composite treatments are equally effective regarding volume reduction of the affected arm. The power calculation was based on an assumption of the relative volume reduction of a minimum 25% and 95% CI bounds of −12 and 12 and thus our 95% CI of −4.30 and −2.26 lies within the range of equivalence. The decrease in volume of lymphoedema (ml) was (mean ± SE): ∆: −31.78 ml (95% CI, −136.96 to 73.40) (Table 2).

**Table 2:** Comparison of changes in volume (primary outcome), excess volume, and circumferences after 1 month and at follow-up (7 months)

	T+MLD		T−MLD		Between-group difference		
	Mean	SE	Mean	SE	Mean	95% CI	*p* Value
At follow-up							
Volume of the affected arm (ml)	2704.3	37.0	2736.1	38.0	−31.8	−137.0; 73.4	0.551
Volume of the affected arm (%)	−6.8	1.2	−5.7	1.2	−1.0	−4.3; 2.3	0.540
Excess volume (ml)	338.1	32.6	310.0	33.5	21.0	−90.2; 132.3	0.708
Excess volume (%)	−31.4	7.4	−35.6	7.6	4.2	−16.7; 25.2	0.687
Circumference of the affected arm (cm)	179.5	0.8	177.9	0.8	1.6	−0.6; 3.9	0.149
After 1 month							
Volume of the affected arm (ml)	2782.9	36.5	2757.8	38.5	25.1	−80.2; 130.3	0.638
Volume reduction of the affected arm (%)	−4.2	1.1	−4.8	1.2	0.6	−2.7; 3.9	0.712
Excess volume (ml)	353.0	32.6	338.4	34.0	21.4	−90.0; 132.7	0.406
Excess volume reduction (%)	−25.3	7.3	−23.4	7.7	−1.9	−22.9; 19.0	0.856
Circumference of the affected arm (cm)	179.2	0.8	178.3	0.8	1.5	−0.8; 3.7	0.196
Circumference of the unaffected arm (cm)	154.4	0.4	153.9	0.4	0.4	−0.6; 1.5	0.425

*SE* standard error, *95% CI* 95% confidence intervals

*p* Value from the superiority trial using a two-sided test hypothesis

In clinical practice, lymphoedema physiotherapists often take the volume measurement of the unaffected arm into account when treating patients with lymphoedema. Since the measurement is a crude estimate, it was not considered suitable as a primary outcome; however, it is an important part of the treatment. Therefore, we also calculated the excess volume. There was no statistically significant difference in the excess volume between groups at 1 month or at follow-up (Table 2). The excess volume decreased significantly in both the groups: T+MLD: 25% reduction at 1 month and 31% reduction at follow-up, T−MLD: 23% reduction at 1 month and 36% reduction at follow-up.

### Secondary outcomes

After the treatment (at 1 month), there was no difference between the groups regarding the circumference of the affected arm (mean ± SE): ∆:1.47 ml (95% CI, −0.77 to 3.70 cm). Likewise, at follow-up, no statistically significant difference was found on the arm circumference between the groups (Table 2). Further, no statistical significant changes were found in volume or excess volume between post intervention (1 month) and follow-up (7 months).

Regarding patient-reported outcomes, the experienced heaviness and tension of the affected arm, shoulder, and chest were evaluated. After treatment, the patients in T+MLD reported a statistically significantly lower experienced tension in the shoulder: ∆: −1.36 (95% CI, −2.38 to −0.35). Other assessments on tension and heaviness showed an insignificant difference between the groups (Table 3). At follow-up, there was no difference between the groups concerning self-reported tension in the arm, shoulder, and chest (Table 3). Additionally, the patients filled out the EQ-5D-5L questionnaire on their status of mobility, self-care, usual activities, and their experience of anxiety and pain. Finally, the patients rated their experienced today’s health on a visual analogue scale. On these variables, no differences were detected between the groups neither at 1 month nor at follow-up (Table 3).

**Table 3:** Comparison of changes in secondary outcomes after 1 month and at follow-up (7 months)

	T+MLD		T−MLD		Between-group difference			
	Mean	SE	Mean	SE	Mean	SE	95% CI	*p* Value
After 1 month								
Heaviness in the arm (scale, 0–10)	2.9	0.4	3.8	0.4	−0.9	0.5	−1.9; 0.1	0.089
Tension in the arm (scale, 0–10)	2.7	0.4	3.5	0.4	−0.8	0.6	−1.9; 0.3	0.137
Tension in the shoulder (scale 0–10)	2.1	0.4	3.5	0.4	−1.4	0.5	−2.4; −0.4	0.009
Tension in the chest (scale, 0–10)	3.5	0.4	3.3	0.4	0.2	0.5	−0.9; 1.2	0.746
Mobility (scale, 0–5)	1.3	0.1	1.5	0.1	−0.2	0.1	−0.5; 0.1	0.149
Self-care (scale, 0–5)	1.4	0.1	1.6	0.1	−0.2	0.1	−0.5; 0.1	0.116
Usual activities (scale, 0–5)	2.0	0.1	2.1	0.1	−0.1	0.2	−0.4; 0.3	0.711
Pain (scale, 0–5)	2.0	0.1	2.1	0.1	−0.1	0.2	−0.5; 0.1	0.370
Anxiety (scale, 0–5)	1.5	0.1	1.3	0.1	0.1	0.1	−0.1; 0.4	0.235
Health today (VAS 0–100)	75.3	2.7	71.2	2.8	4.1	3.9	−3.6; 11.7	0.292
BMI (kg/m^2^)	29.2	0.1	29.2	0.1	−0.0	0.2	−0.3; 0.3	0.972
At follow-up								
Heaviness in the arm (scale, 0–10)	3.6	0.4	3.5	0.4	0.1	0.5	−1.0; 1.1	0.923
Tension in the arm (scale, 0–10)	3.5	0.4	3.1	0.4	0.4	0.6	−0.7; 1.4	0.512
Tension in the shoulder (scale 0–10)	2.7	0.4	2.7	0.4	−0.1	0.5	−1.1; 1.0	0.919
Tension in the chest (scale, 0–10)	3.6	0.4	3.1	0.4	0.5	0.5	−0.6; 1.5	0.393
Mobility (scale, 0–5)	1.5	0.1	1.6	0.1	−0.2	0.1	−0.4; 0.1	0.285
Self-care (scale, 0–5)	1.2	0.1	1.4	0.1	−0.2	0.1	−0.5; 0.1	0.092
Usual activities (scale, 0–5)	2.0	0.1	2.1	0.1	−0.1	0.2	−0.4; 0.3	0.623
Pain (scale, 0–5)	2.0	0.1	2.2	0.1	−0.2	0.2	−0.5; 0.1	0.315
Anxiety (scale, 0–5)	1.4	0.1	1.4	0.1	−0.1	0.1	−0.2; 0.2	0.876
Health today (VAS 0–100)	74.4	2.7	74.7	2.8	−0.3	3.9	−8.0; 7.3	0.931
BMI (kg/m^2^)	29.2	0.1	29.4	0.1	−0.2	0.2	−0.5; 0.1	0.234

*BMI* body mass index, *SE* standard error, *95% CI* 95% confidence intervals

*p* value from the superiority trial using a two-sided test hypothesis

A comparison between our study group and Danish EQ-5D population norms^19^ stratified on age groups 20–29, 30–39, 40–49, 50–59, 60–69, and 70–79 show that our study group scores were significantly lower compared to the Danish population norms for the EQ-5D index score stratified on age groups <60 years (all *p* < 0.005) but not in the decades of the two senior ages. The number of patients below the age of 50 years were 32 and 38 patients were ≥ 60 years.

The majority of the entire group of patients or 61% (*n* = 47) reported daily use of the sleeve in the follow-up period. Nine percent (*n* = 7) used it on average >3 times per week, 5% (*n* = 4) ≤1 time weekly, and 2 patients did not use the sleeve at all. Seventeen patients (*n* = 17) did not answer the question regarding the use of the sleeve.

### Post hoc analyses

We performed paired analyses on the self-reported outcomes in each group and found a statistically significant reduction in the experienced heaviness of the affected arm: mean change of 1.4 (95% CI, −2.3 to −0.6; *p* = 0.001), tension in the arm; mean difference of −1.4 (95% CI, −2.3 to −0.5; *p* = 0.003) in T+MLD, 1 month after treatment. The results on tension in the shoulder and chest were insignificant with *p* = 0.072 and *p* = 0.056, respectively. Paired analyses on these variables in T+MLD from baseline to follow-up were not statistically significant. In T−MLD, the results from baseline to 1 month showed statistically significant results on the experienced tension in the arm, mean difference of −1.1 (95% CI, −1.9 to −0.2; *p* = 0.020), and tension in the chest, mean change −1.3 (95% CI, −2.1 to −0.4; *p* = 0.005), but not regarding the experienced heaviness of the arm (*p* = 0.080) and tension in the shoulder (*p* = 0.595). At follow-up, T−MLD had significant results in all variables (all *p* < 0.008) apart from tension in the shoulder (*p* = 0.166).

We also conducted paired analyses on the health-related outcomes. T+MLD reported less problems with usual activities (*p* = 0.014) after 1 month and T−MLD reported less pain (*p* = 0.042), but there were no statistically significant results in the other outcomes. At follow-up, none of the health-related outcomes improved significantly.

## Discussion

This study shows that physiotherapeutic treatment of lymphoedema among breast cancer survivors significantly reduces the volume of lymphoedema; however, neither one of the two treatments tested in this study is superior to the other. When analysed in each group separately, both treatments showed positive effects on self-reported outcomes; however, no between-group differences were detected at follow-up. According to the sample size calculation, the range of −12 to 12 was predefined as an acceptable range for imprecision. Since our results shows 95% CI boundaries of −4.3 to 2.3 on differences between the groups on primary outcome, we are in a way able to demonstrate that treatment without MLD is no worse than treatment with MLD. Unfortunately, with respect to the power calculation, insufficient number of patients were included and therefore we cannot exclude the risk of Type 1 error, i.e. incorrect rejection of the null hypothesis. A null hypothesis in a equivalence study like this will state that ‘new’ treatment (without MLD) was not as effective as the standard treatment (with MLD).^22^ However, of note, power calculations are an estimation based on assumption, which, if incorrect or unknown, will lead to inaccurate estimates.

The scientific evidence regarding the effect of MLD is not convincing and other randomised controlled trials aiming to measure the effect on arm volume, when adding MLD to compression bandaging, have also shown ambiguous and insignificant effects.^12,13^ Some of these trials, focussing on MLD+bandaging vs. bandaging alone in order to reduce lymphoedema volume, found results more or less similar to ours.^10,11,20,21^ We think that lymphoedema volume is an appropriate primary outcome since the volume of lymphoedema causes an experienced heaviness and tension of the arm and affects the quality of life and sensation of pain. There is some divergence in how to measure the volume of lymphoedema, and according to review on MLD for lymphoedema following breast cancer treatment (Cochrane Library), there is a need for a clinically meaningful volumetric measurement.^13^ We appreciate the method converting circumference measurements into volume^23^; however, we argue that measuring the lymphoedema volume by the Inverse Water Displacement Volumetry method minimises the risk of measurement errors compared to adding up several circumference measurements. For the same reasons, we did not use the excess volume as the primary outcome since this measurement may induce errors. On the other hand, the purpose of the present study was to design a study illustrating our clinical practice by using a treatment method and a volume measurement similar to the methods we offer our patients. Measuring the circumference and excess volume, with the healthy arm included in the calculation, is a part of the clinical practice and, therefore, also used as an outcome measurement in this study. The lymphoedema volume may be affected by seasonal variations, body weight, etc. We included patients at all seasons of the year; however, we argue that the randomisation ensures even allocation and a fair comparison between the groups regarding possible differences explained by variation in the weather, normal fluctuations in the limb volume, etc. We measured the body weight three times during the study. There were no changes in body weight during the study either within groups or between groups.

Over the years, the CDT has been based on treatment 4–5 times weekly where each treatment takes 45–60 min. This also applies MLD treatment by Földi^24^ or Vodder.^25^ Several studies include MLD treatment 4–5 times a week,^10,11,20,26^ but it is uncertain whether the number of treatments directly reflects the volume measurement. We found no studies testing the most optimal number of treatment. We designed an intervention consisting of MLD treatment only twice a week for 30 min. It may be argued that treatment twice a week does not reflect the principles for CDT and thus is not sufficient to achieve a superior effect. Our motive for our design was the experience that standardisation among different treatment locations and among treating physiotherapists was lacking, and in the clinical practice at some hospitals, the number of treatments were reduced anyway because of the improved bandages. In addition, our study shows that, regarding the volume, treating lymphoedema without MLD is as effective as adding MLD to the treatment when offered twice a week. It is not likely that bandaging will be excluded from the treatment, thus the remaining questions are, would additional MLD treatments per week lead to further volume reduction and, if yes, to what extent? Mean reduction in lymphoedema excess volume varies somewhat among studies and even though the studies have different designs in content and number of treatments, the mean reduction is often compared. Without taking these differences into account, we consider that a reduction of approximately 31% in lymphoedema in the present study is a reasonable effect. McNeely et al. report 46% reduction post intervention^20^ and Johansson and colleagues report 15% reduction.^26^ Both studies used volume displacement to measure the lymphoedema volume. Bergmann et al., Dayes et al., and Andersen et al. report 26, 29, and 43% volume reduction, respectively, and these studies applied formula to calculate lymphoedema volume from circumferential measurements.^10,21,27^

Most RCTs on lymphoedema are relatively small studies. It could be due to strict inclusion criteria. The inclusion criteria differ slightly among the studies,^10,21,28^ but overall, our study is comparable with respect to the inclusion criteria in the mentioned studies. Some studies use a minimum limit of percentage increase in the arm volume as an inclusion criterion. In our study, the measurement of 2 cm difference in circumference between the affected and unaffected arm was one of the inclusion criteria and the percentage increase was not calculated. This may have caused relatively larger number of patients with mild lymphoedema included in our study. We calculated the percentage subsequently, showing that 50% of the patients had mild lymphoedema. All in all, the symptom burden was low, function as per EQ-5D high—consistent with mild lymphoedema. By strictly adhering to the inclusion criteria, we may have contributed to an extended inclusion period. Long inclusion periods can be a methodological issue, since unforeseen changes may occur for the duration of that period. Many patients were also referred earlier to lymphoedema treatment because of the fast track cancer referral programme in Denmark. These patients did therefore not fulfil this study’s inclusion criteria of minimum 2 cm difference in circumference, which challenged the enrolment. The patients in the present study achieve a positive effect of the treatment regardless of allocation and 57% of the study population had mild lymphoedema. It has been discussed whether patients with mild lymphoedema to a greater extent benefit from MLD compared to patients with moderate/severe lymphoedema^20^; however, this issue has not been explored yet and, therefore, cannot be excluded.

We were not able to show any differences in self-reported outcomes at follow-up. The questionnaire used to evaluate health outcomes, EQ-5D-5L, has been translated into >170 languages; however, ceiling effects have been reported and it can be discussed if EQ-5D-5L is an obvious choice of questionnaire to the group of breast cancer patients. We compared the study group in this study to Danish population norms,^19^ and the comparison showed that women aged >60 years did not differ significantly from the population norms, whereas the younger women did. The reason could be that younger women assess their breast cancer diagnosis as being more radical in relation to their general status of daily activities than older women who may already have some limitations regarding mobility, self-care, and usual activities. Since the mean age of our study population was 61 years, a more sensitive questionnaire about the health outcome may have been preferable.

Unfortunately, we did not reach the required sample size of 76 in each group or a total of 152 patients. This may reduce the weight behind the statement that treatment without MLD is sufficiently similar to the treatment where MLD is included. On the other hand, one of the prerequisite for the sample size calculation was 95% CI bounds of −12 and +12% for the mean difference and a significance level of 0.05, assuming a null difference. The study shows that between-group difference on the primary outcome was (mean): ∆ −1.0, 95% CI: −4.3 to 2.3%—indicating that equivalence may be concluded.

### Strengths and limitations

The study design and the stringency are the main strengths of this study. Other strengths are the use of objective volume measurements and the emphasis on patient-reported outcomes. The design of the Bravometer used to measure the lymphoedema volume prevented us to measure the arm and the shoulder concurrently. This may be a limitation since the shoulder might be affected by the lymphoedema. On the other hand, measuring circumferences, as is done in the clinic, does not include the shoulder either.

Retrospectively, it would have been more appropriate to select a primary outcome as lymphoedema volume expressed as ml rather than percentage. Our choice was based on the existing literature in the field. Overall, it was challenging to get all required information about minimal clinical and statistical difference in published studies on lymphoedema treatment due to heterogeneity in study design and quality. Ōwing to slow recruitment, the trial terminated before a sufficient number of patients were included according to the power calculation, which is a limitation resulting in a low statistical power.

## Conclusion

This trial suggests that treatments without MLD are comparable in effectiveness to treatments with MLD in terms of lymphoedema volume reduction. These results are in line with several other studies. It is still not clear whether the MLD among certain groups of breast cancer patients should be part of the treatment or whether MLD is effective without bandaging. The results emphasises an implementation and dissemination of evidence-based treatments of lymphoedema in the clinical practice.
